# Effects of intervertebral disc disorders of low back on the mandibular kinematic: kinesiographic study

**DOI:** 10.1186/1756-0500-7-569

**Published:** 2014-08-26

**Authors:** Alessandro Spadaro, Irma Ciarrocchi, Chiara Masci, Vincenzo Cozzolino, Annalisa Monaco

**Affiliations:** Department of Life Health and Environmental Sciences, University of L’Aquila, L’Aquila, Italy; Osteopathic Manipulative Medicine. Department of Life Health and Environmental Sciences, University of L’Aquila, L’Aquila, Italy; Department of Life Health and Environmental Sciences, University of L’Aquila, via Vetoio, Coppito, 67100 L’Aquila Italy

**Keywords:** Intervertebral disc disorders, Kinesiography, Mandibular kinematics, Sympathetic nervous system

## Abstract

**Background:**

Intervertebral disc disorders are one of the most common causes of low back pain. Neuromuscular dysfunction frequently is present in patients with lumbar disc herniation.

When considering joint dysfunction, it is important to remember that the spine functions as a unit. Dysfunction on one level can trigger compensatory changes in other spinal levels or in other areas of the musculoskeleton. Findings demonstrated the relationship between stomatognathic and postural systems justifying the hypothesis that muscular-skeletal impairment in one system could affect the other one. However, evidence that a lumbar intervertebral disc herniation could influence the mandibular kinematics is still lacking. Aim of this study was to analyse the effects that intervertebral disc herniation of low back could have on the mandibular kinematics.

**Findings:**

Kinesiographic evaluations of the mandibular dynamics of 23 adult patients suffering L4/L5 and L5/S1 lumbosacral disc hernation were compared with a non pathological control group. A statistically significant difference of maximal mouth opening (*p <* .05) and of maximal mouth opening velocity (*p <* .03) was found comparing the study patients with the control subjects.

**Conclusion:**

Lumbosacral disc herniation appears to be associated with changes in the activity of mandibular kinematics both in rate and quality of movement. The study suggests the existence of connections between masticatory system and lumbar disk herniation.

## Background

Some Authors discussed the relationship between stomatognathic and postural systems [1, 2].

Clark et al. [3] showed co-activation of sternocleidomastoid and masseter muscles. Trigeminal electrical and mechanical stimulation elicited sternocleidomastoid inhibition showing functional coupling between mandible and neck-trunk system. Ehrlich et al. [4] supported Clark’s data stating that sternocleidomastoid, trapezius, paravertebral and rectus abdomis muscles increased from 3.3 to 7.6 times their Surface Electromyography (sEMG) resting activity during clenching [5, 6].

According to Giannakopoulos et al. [6] there is a close association between the head and neck movements.

Trigeminal nerve has numerous neuroanatomical connections within the brainstem and several projections to all levels of the spinal cord. This leads a variety of neuromuscular interactions, for instance, synchronized extension–flexion movements of the head during jaw-opening/closing cycles [7].

Head position is an important factor in determining the amount of vertical mandibular opening in healthy adults. Higbie et al. [8] stated that vertical mandibular opening ranged from 44 mm to 36.2 mm changing from extended to flexed head position. In a recent work Monaco et al. compared Osteophatic Manipulative Therapy (OMT) effects on two groups of subject affected by temporomandibular disorders (TMD): study group and no-intervention group. The study group, treated with OMT of postural system, showed a significant improvement of maximal mouth opening and maximal mouth opening velocity compared with no-intervention group indicating that manipulative treatment of no-stomathognatic areas was related to changes in the kinesiographic (KNG) activity of mandible [9].

Intervertebral disc disorders (IVDs) is one of the most common causes that lead to low back pain.

The lifetime prevalence of symptomatic herniated disks is estimated at 1% to 3% [10, 11], although anatomic evidence of disk herniation has been found in 20% to 40% of imaging tests among asymptomatic people [12, 13]. Most clinically relevant herniations occur between the ages of 30 and 50 but can also occur in adolescents and older people.

Neuromuscular dysfunction frequently is present in patients with lumbar disc herniation [14]; in patients with lumbar disc herniation, muscle strength of the trunk and knees was decreased to a similar extent [15].

When considering joint dysfunction, it is important to remember that the spine functions as a unit. Dysfunction on one level can trigger compensatory changes at other spinal levels or in other areas (leg, hip, knee, ankle) of the musculoskeleton [16].

Findings demonstrated the relationship between stomatognathic and postural systems justifying the hypothesis that muscular-skeletal impairment in one system could affect the other one.

Several authors have highlighted the importance of electromyographic and kinesiografic analysis in the assessment of Stomatognathic System and mandibular kinematics [17–22].

However, evidence that a lumbar intervertebral disc herniation could influence the mandibular kinematics is still lacking. Hence the aim of this study was to investigate the mandibular kinematics by using kinesiographic instruments in adult patients with lumbosacral disc herniations to compare these data with those of the non-pathologic control subjects. This could be a great interest for the researchers, as it could contribute to clarify the nature of the relationship between body posture and stomatognathic apparatus.

## Findings

Twenty three subjects, Caucasian adults (average age 35 yrs SD, i.e. Standard Deviation, 8.6 yrs) presenting L4/5 or L5/S1 disc herniations (disk protrusion according to a morphological classification of disk herniation) [23] diagnosed by clinical and radiographical evaluation at least 6 months before testing, and an equal number of voluntary control subjects (average age 36.6 yrs SD 7.9 yrs), matched for age and sex (14 male and 9 female), with no pathology of the intervertebral disc, were included into the sample.

The patients were selected from an initial group of 65 patients with intervertebral disc herniation from the Unit of Physiatry, University of L’Aquila, based on the following criteria, which were also used to select the control subjects: (a) absence of any previous orthodontic treatment; (b) presence of full natural permanent dentition (28 teeth at least) and a bilateral molar support with molar and cusp Angle class I; (c) Normal facial type (d) absence of cross-bite, (e) absence of dental restorations that might alter dimensions, shape, and position of the mid-point of the 161 clinical crown (f) no prosthetic rehabilitation (g) absence of previous surgical treatments of the affected disc; (h) no missing teeth (with the exception of the third molars) (i) absence of periodontal problems, (l) absence of low back pain (m) lack of Temporo-mandibular joint pain (n) absence of treatment for TMD.

Exclusion criteria were (a) skeletal anomalies (b) malocclusions, (c) painful dysfunction of the cranio-cervical region (d) presence of carious teeth; (e) presence of prosthetic rehabilitation; (f) presence of a unilateral or bilateral cross-bite; (g) trauma in the dental-facial region; (h) skeletal asymmetry; (i) genetic or congenital anomalies;

Ethics approval was obtained by the University’s Review Board for Health Sciences Research involving Human Subjects, and all subjects were provided of written-informed consent before testing.

### Kinesiographic equipment and measurements

**Figure 1:**
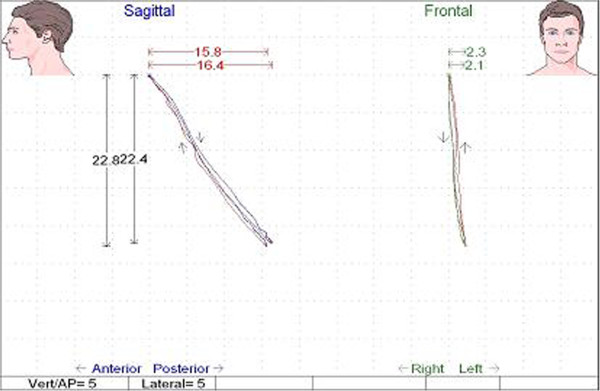
**Kinesiographic track showing the amount of opening movement (mm) of study group patient in the two traces (sagittal and frontal).**

**Figure 2:**
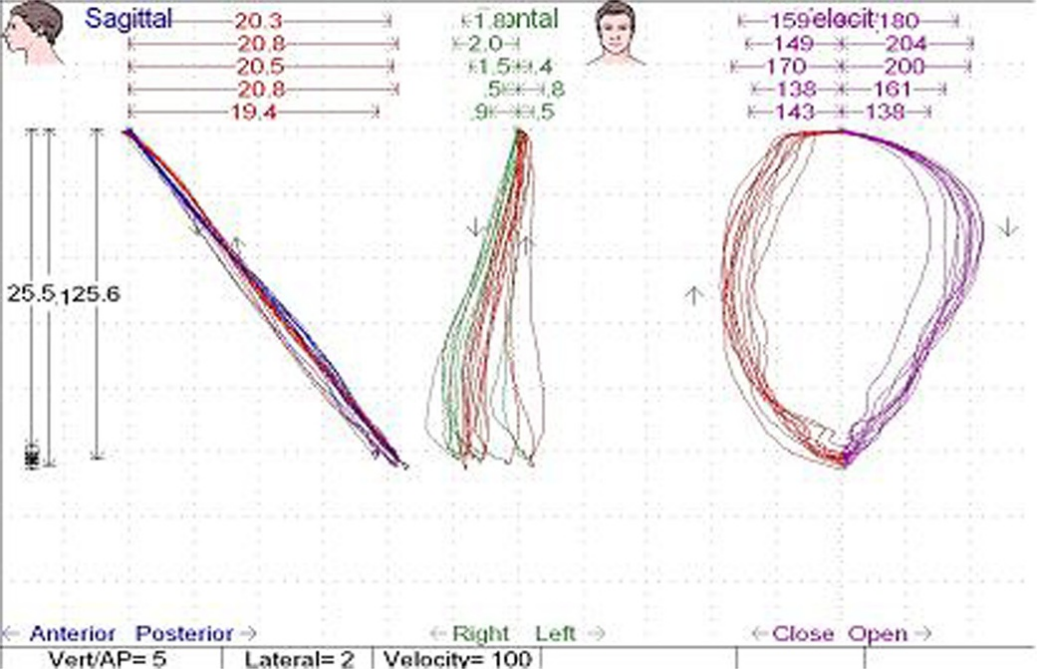
**Kinesiographic track showing the amount of opening-closing movement velocity (mm/s) of study group patient in the three traces (sagittal, frontal and velocity).**

Mandibular movements were recorded by the kinesiograph of the K7 Diagnostic System (Myotronics Research Inc., Seattle, WA, USA). The equipment consisted of an array of sensors placed on the subject’s head that provided information about the position of the mandible. When the mandible moved, changes in the magnetic flux of the small bar magnet fixed on the mandibular incisor teeth were detected. The kinesiograph was connected to a computerized system that recorded and displaied spatial coordinates in the vertical, antero-posterior and lateral axes to the nearest 0.1 mm. For each movement the software package indicated the amount and the velocity of the movement. The measures were in millimeter for the amount of movement and in mm/sec. for the velocity (Figures 1 and 2).

The sample rate was 125 Hz; during every single second 125 samples were collected by the kinesiograph regarding position of the magnet and velocity of its movement.

During the recordings the patient was comfortably seated in a wood chair with headrest, placed in a comfortable room, with eyes closed to avoid environmental information.

Patients were previously informed on the movements they had to do; the recording session started only when the patient understood the correct way to perform the kinesiographic tests.

In the first test the patients were asked to open the mouth as wide as possible starting from occlusal contact. The operator stopped them after the recording of 3 consecutive movements.

In the second test the subjects opened the mouth as fast and wide as possible, reaching the maximal opening position and, finally, to close the mouth returning to occlusal contact. After 15 successive movements the operator stopped the patients. Each kinesiographic trial was provided of two KNG recordings. This record allowed to obtain the following parameters:

Maximal Opening Velocity (MOV): it is the maximal velocity reached during the movement of opening starting from occlusal position (velocity = 0 mm/sec.) and ending in maximal opening position (velocity = 0 mm/sec.).

Maximal Closing Velocity (MCV): it is the maximal velocity reached during the second phase of the opening/closing movement staring from maximal opening position (velocity = 0 mm/sec.) and ending to occlusal position (velocity = 0 mm/sec.).

The opening movements were performed up before pain onset.

20 seconds passed between the two recordings.

The patient was accepted if kinesiographic values didn’t exceed 1 standard deviation (no missing patient was observed).

KNG tracks were examined by a second operator without knowledge of recording purpose.

### Statistical analysis

We have calculated that 23 patients per group was sufficient to demonstrate a statistically significant difference (α = 0.05) of 5 mm in the mouth opening between the study group and the control group, with a statistical power (1-β) of 90%.

A Student’s t-test for independent samples was performed, using Stata statistics Package, on means and variance values of kinesiographic data to assess the significance of the differences in KNG activities between the study and the control group. People with lumbar disk herniation formed the study group, people without lumbar disk herniation represented the control group.

Differences with a value of *p* < .05 and < .005 were respectively regarded as significant and highly significant.

In null hypothesis no significant difference in means and variance shows that the two group have the same mandibular kinematics and the lumbar herniation probably doesn’t influence the characteristics of the mandible movement.

In alternative hypothesis significant difference could show that lumbar disk herniation could affect the mandible kinematics.

Table 1 shows Mean values and Standard Deviation (in parenthesis) of kinesiographic data of study and control group.

**Table 1:** **Mean values and standard deviation (in parenthesis) of kinesiographic data of study and control group**

PARAMETER (m.u.)	CONDITION	MEAN (S.D.)
**MO** (mm)	SG	34.77 (6,44)
	CG	41.94 (2,72)
	Diff.	.05*
**MOV** (mm x s^−1^)	SG	244.8 (109,3)
	CG	321.3 (85,1)
	Diff.	.003**
**MCV** (mm x s^−1^)	SG	290.0 (108,4)
	CG	320.0 (92,3)
	Diff.	NS

**MO** = Maximal Opening; **MOV =** Maximal Opening Velocity; **MCV** = Maximal Closing Velocity; m.u. = measure unit;

* **=** significant; ** = Highly significant; NS = Not Significant; SD = standard deviation.

In our study patients who suffered disc herniation showed both a lower amount of vertical mandible maximal opening (MO) and a lower maximal opening velocity (MOV) compared to control group.

Dysfunction on one level can trigger compensatory changes at other spinal levels or in other areas (leg, hip, knee, ankle) of the musculoskeleton [16].

In describing these relations, McAndrews [24] used the artful metaphor of a mobile hanging over a child’s crib. When one of the mobile’s strings is cut, all of its suspended ornaments start to bounce and shift erratically until achieving a new equilibrium. In this new state of equilibrium, however, the ornaments have shifted both in relation to the central axis and in relation to each other. The body’s musculoskeletal system works in much the same way. When equilibrium is disrupted, whether by injury, chronic postural stress, or other causes, structural patterns are altered to a greater or lesser degree depending onto the nature and intensity of the forces that threw off the old pattern of balance. Over time compensatory imbalances can embed themselves deeply as muscles, ligaments, cartilage, and even bone undergo changes in structure and function. The result could be a chronic musculoskeletal imbalance and pain. A key corollary of the principle of compensation is that the site of pain may not be the site of the pain’s cause. For instance, some cases of knee pain result from structural injury to the knee while others are compensations for mechanical joint dysfunction in the lumbar spine or sacroiliac joints.

A consequence of the longitudinal organization of central nervous system (CNS) is that a lesion in a lower district could influence upper levels of structural organization, by determining postural adjustments in relation to somatosensorial information changes [2, 25].

The recent findings of Monaco et al. [9] cited in the introduction supported the Irvin data showing that the established postural homeostasis with OMT resulted in improvement of mandible kinetics, in particularly in MO and in MOV [26].

One of the reasons asserted to explain results obtained on mandible Kinematics by OMT was that direct and indirect sympathetic control could affect some muscular-skeletal symptoms, including restricted range of active and passive movement or pain.

Various coupling and regulating mechanisms have been proposed to explain the homeostatic influence on physiologic processes responsible for maintaining restricted range of movement and pain [27–29]. Homeostasis may be altered through sympathetic, biochemical or neuroendocrine mechanisms affecting specifying structures or target receptors, or both.

Modulation of sympathetic tonus, enhancing healing rates, has been linked to improvement of visceral and, in the light of our study, somatic functions [30].

As claimed by osteopathic literature osteopathic lesion, responsible of movement restriction, is referred to impairment of sympathetic transmission [9]. According to this hypothesis manipulative treatment enhancing balance in sympathetic nervous system could improve movement restriction.

Somatomotor system and sympathetic nervous system (SNS) are intimately correlated. SNS supplies motor performance by modifying vegetative function parameters to meet the varying metabolic requirements of the active muscle [31, 32]. Increase in SNS outflow affects motor function through actions exerted at the muscle level.

Passatore et al. [31] stated that sympathetic nervous system controls both muscle blood flow and intracellular contractile mechanism and may affect motor function by modulating afferent activity from muscle spindles that are highly concentrated in jaw-closing muscles. In his study the electrical stimulation of rat sympathetic superior ganglion (SSG) cause an impairment of jaw jerk reflex.

Recent immunohistochemical data on masseter muscle confirmed the presence of non vascular sympathetic innervation on muscle spindles in close association with intrafusal muscle fibers [33]. These data support Passatore data and suggest that sympathetic nervous system could modulate the spindle afferent discharge by altering intrafusal fiber mechanics [33]. Increase in SNS outflow may act by: 1. decreasing muscle blood perfusion, which is an inseparable factor of muscle pain; 2. enhancing contractile force in fast-contracting muscle, while exerting a fatiguing action on slow-contracting ones; 3. reducing the quality of proprioceptive information. The latter action is likely to worsen different aspects of motor control increasing of co-contraction of antagonist muscles aimed at recovering movement precision by increasing joint stiffness. This effect has been studied in “in vitro” and “in vivo” and seems to be particularly powerful in jaw closing muscles [34].

Koolstra et al. [35] demonstrated in open jaw movement the passive forces produced by the jaw-closing muscles were remarkably stronger than those produced by the jaw-opening muscles in close jaw movement.

The foregoing statements could explain the findings of our study on MO and MOV. First of all the different but impaired mandible kinematics showed by people suffering IDVs compare to control group clearly confirmed the functional and the dysfunctional relationship among different part of the body quoted in literature, even when the two part aren’t contiguous like low-back and mandible are. It’s possible that only the connectivity of Nervous System could explain this relationship.

On the other end considering the stronger resistance exerted by closing jaw muscles (masseter, anterior temporalis and medial pterygoid) on opening movement it is possible that the effect of hyperactivity of Sympathetic Nervous System could be remarkably higher in these muscles. In this case the most influenced movement of the mandible would be the opening movement in which, according to co-activation of closing and opening muscles, closing muscles resist to the movement. Our data on MO and MOV, maximal opening movement and maximal velocity of opening movement, could confirm previous cited findings.

A limit of our work is that this is a cross sectional study, so it is not defined the actual timing of the problems. Ideally, a study should be conducted following patients over several years, investigating patients before low back problems and during their disease and treatment. This type of longitudinal study is in progress in our clinic, but the results will not be available for several more years.

## Conclusions

This preliminary study compared Kinesiographic data of patients affected by Intervertebral disc herniation with those of control subjects. The patients showed a significant reduction of maximal mouth opening and maximal opening velocity compared with the control group.

Findings in our study allow hypothesising active and direct involvement of sympathetic nervous system on stomatognathic kinematics. Considering the close anatomical relation between sympathetic cervical system, which supplies facial and cranial districts, and cervical spine and, through it, head, neck and trunk posture, it is possible to suggest the critical role in relate stomatognathic and postural system performed by sympathetic nervous system.

Future investigation will be aimed at evaluating these variables in a longitudinal model and to evaluate them before and after the disc herniation resolution in order to clarify the mechanism at work.

## Abbreviations

sEMG: Surface electromyography
TMJ: Temporomandibular joint
OMT: Osteophatic manipulative therapy
TMD: Temporomandibular disorders
KNG: Kinesiographic
IVDs: Intervertebral disc disorders
MO: Maximal opening
MOV: Maximal opening velocity
MCV: Maximal closing velocity
SNC: Central nervous system
SNS: Sympathetic nervous system
SSG: Sympathetic superior ganglion.
